# Partial-brain radiotherapy for primary central nervous system lymphoma: multi-institutional experience

**DOI:** 10.1093/jrr/rrv085

**Published:** 2015-12-09

**Authors:** Michio Iwabuchi, Yuta Shibamoto, Chikao Sugie, Shiho Ayakawa, Hiroyuki Ogino, Fumiya Baba

**Affiliations:** 1 Department of Radiology, Nagoya City University Graduate School of Medical Sciences, 1 Kawasumi, Mizuho-cho, Mizuho-ku, Nagoya, Aichi, 467-8601, Japan; 2 Japan Community Healthcare Organization Chukyo Hospital, 1-1-10 Sanjyou, Minami-ku, Nagoya, Aichi, 457-8510, Japan; 3 Nagoya Proton Therapy Center, 1-1-1 Hirate-cho, Kita-ku, Nagoya, Aichi, 462-8508, Japan; 4 Nagoya City West Medical Center, 1-1-1 Hirate-cho, Kita-ku, Nagoya, Aichi, 462-8508, Japan

**Keywords:** primary central nervous system lymphoma, PCNSL, radiation therapy, partial-brain radiotherapy, neurocognitive function

## Abstract

Whole-brain radiotherapy (WBRT) has been an important component of treatment for primary central nervous system lymphoma (PCNSL), but delayed neurotoxicity has been a matter of concern. We have employed partial-brain radiotherapy (PBRT) with wide margins for PCNSL patients with a single lesion or a few lesions. In this study, we evaluated the treatment outcome in PCNSL patients undergoing PBRT. Between 2003 and 2014, 24 patients were treated with PBRT; 16 received high-dose-methotrexate (MTX) –containing chemotherapy before PBRT. Conventional fractionation with a median dose of 54 Gy was used. For reference, 15 patients undergoing MTX-based chemotherapy and WBRT were also analyzed. The 3-year overall survival rate was 60% for all 24 patients undergoing PBRT and 68% for the 16 patients undergoing MTX-based chemotherapy plus PBRT. The 3-year progression-free survival rate was 41% for all 24 patients undergoing PBRT and 36% for the 16 patients undergoing MTX-based chemotherapy. The in-field recurrence rate was 26% and the out-of-field recurrence rate was 15% at 3 years for all 24 patients undergoing PBRT. The rates for in-field recurrence and the out-of-field recurrence were 27% and 21%, respectively, for the 16 patients undergoing MTX-based chemotherapy. CNS-recurrence rates were similar in patients undergoing MTX-based chemotherapy and PBRT to the rates in those undergoing MTX-based chemotherapy and WBRT. Neurocognitive dysfunction developed in 3 of the 16 patients undergoing MTX + PBRT and in 4 of 15 patients undergoing MTX + WBRT (*P* = 0.68). PBRT seems to be a feasible treatment option for solitary PCNSL. Further investigations are warranted to evaluate the advantages of PBRT over WBRT.

## INTRODUCTION

Until the late 1970s, the standard treatment for primary central nervous system lymphoma (PCNSL) was whole-brain radiation therapy (WBRT). Although most PCNSLs responded to WBRT, recurrence often developed and the patients had a poor prognosis, with reported 5-year survival rates usually <10% [1–3]. Thereafter, the use of systemic chemotherapy was attempted. While chemotherapy regimens like CHOP (cyclophosphamide, doxorubicine, vincristine and prednisolone) showed limited efficacy [4, 5], high-dose-methotrexate (MTX) –containing regimens appeared more effective, and they led to an improvement in the prognosis [6–8].

MTX-containing chemotherapy appeared effective in achieving long-term survival, but a considerable number of patients, especially elderly patients, developed neurocognitive dysfunction when it was combined with WBRT [9–11]. Therefore, chemotherapy-alone approaches were attempted, but it was recognized that the omission of radiation therapy was associated with increased recurrence [12–14]. Based on these experiences, neuro-oncologists recently advocated the appropriate use of radiation therapy in the treatment of PCNSL [14, 15].

In using radiation therapy for PCNSL, two approaches are conceivable for reducing the adverse effects of standard-dose WBRT (40–50 Gy). One is to use reduced doses, and this is being tested [14, 16]; some results will be obtained in the near future. Another is to use a reduced volume of radiation therapy. Shibamoto *et al.* [17] advocated the use of partial-brain radiation therapy (PBRT) with generous margins for PCNSL. This was considered a reasonable approach when combined with high-dose-MTX–based chemotherapy. Based on their reports, our group has used PBRT for PSNCL with a single or a few contrast-enhancing lesions. In this study, we retrospectively analyzed the outcome of PCNSL patients undergoing PBRT with or without MTX-based chemotherapy. For reference, we also analyzed 15 patients treated with WBRT plus MTX-based chemotherapy.

## MATERIAL AND METHODS

### Patients

Between 2003 and 2014, 24 patients (median age, 66 years; range, 22–90 years) with pathologically confirmed PCNSL were treated with PBRT with or without chemotherapy at Nagoya City University and three affiliated hospitals. These hospitals are closely related to each other, so the treatment policy is identical among these institutions. Informed consent had been obtained from each patient prior to radiotherapy. No patients had an elevated human immunodeficiency virus titer. This retrospective study was approved by the Institutional Review Board. For reference, 15 patients with pathologically confirmed PCNSL treated with WBRT and MTX-based chemotherapy during the same period (2003–2014) were also analyzed.

The patient, tumor and treatment characteristics are summarized in Table 1. MTX-based chemotherapy was used in 16 of the 24 patients in the PBRT group, and they were separately analyzed. Of the 24 patients undergoing PBRT, multiple tumors were seen in 4 patients (17%). The median maximum tumor diameter was 37 mm (range, 12–70), and the median radiation dose was 54 Gy.

**Table 1.** Patient characteristics and radiation dose

	PBRT	PBRT + MTX	WBRT + MTX	*P*
Patient number	24	16	15	
Age: median (range)	66 (22–90)	68 (58–78)	60 (22–69)	0.71
Sex: male/female	10/14	5/11	9/6	0.10
Performance status: (0–2/3,4)	15/9	10/6	11/4	0.51
Pathological subtype: Diffuse large B-cell/	18/0/6	14/0/2	12/2/1	0.57
Peripheral T-cell/NHL (unspecified)				
Tumor number: single/multiple	20/4	14/2	3/12	0.001*
Maximum tumor size (mm): median (range)	37 (12–70)	38.5 (12–60)	32 (13–61)	0.22
LDH elevation: yes/no	5/19	5/11	5/10	0.90
Radiation dose (Gy): median (range)	54 (30.6–64.8)	50 (30.6–54)	40 (23.4–50)	0.01*

NHL = non-Hodgkin's lymphoma, MTX = methotrexate, LDH = lactate dehydrogenase.

### Radiation therapy

All radiation therapy was used as the first line treatment. In principle, PBRT was used for a single PCNSL lesion and multiple contrast-enhancing lesions; most of the latter were considered contiguous. Otherwise, WBRT was considered. WBRT was also delivered according to the request of an attending neurosurgeon or medical oncologist regardless of the tumor number. Radiation therapy was delivered using 6- or 10-MV X rays from linear accelerators. The volume for PBRT included the tumor mass plus 4-cm or larger margins. All high-intensity areas on T2-weighted images were included. For large tumors, the treatment volume became considerably large, but some parts of the peripheral normal brain tissues were still out of the treatment volume. The rotational, 4-field box, or parallel opposing techniques were used for PBRT, depending on the tumor location. Radiation therapy was given five times a week, and daily doses of 1.8–2 Gy were used. After the PBRT or WBRT, the radiation field was reduced after 30–40 Gy to give a focal boost that covered the original gross tumor volume plus 1-cm margins whenever necessary.

### Chemotherapy

Chemotherapy was used at the discretion of the attending neurosurgeons or medical oncologists. All chemotherapy was performed prior to radiation therapy. For the 24 patients undergoing PBRT, a MTX-based regimen was considered, but 8 patients refused or were judged to be unsuitable for the chemotherapy. So, 5 of them received four or five courses of CHOP or rituximab–CHOP, and 3 received no chemotherapy. The MTX-based regimens were high-dose MTX (3.5 g/m^2^) alone in 9 of the 24 patients, MTX (3.5 g/m^2^) plus rituximab (375 mg/m^2^) in 6 patients, and MTX (3.5 g/m^2^) with rituximab (375 mg/m^2^), procarbazine (100 mg/m^2^) and vincristine (1.4 mg/m^2^) in 1 patient. Three to five courses were administered.

For the 15 patients undergoing WBRT plus MTX-based chemotherapy, 8 received MTX alone and 7 received MTX in combination with rituximab (375 mg/m^2^), procarbazine (100 mg/m^2^) and vincristine (1.4 mg/m^2^). Three to five courses were administered.

### Follow-up evaluation

All patients were followed at our institutions until the terminal stage or at least for some while, so data on recurrence pattern and post-irradiation status until last follow-up were available. Tumor response after radiation therapy was evaluated at the time of maximal tumor regression based on the response evaluation criteria in solid tumors. Recurrence was considered ‘out of field’ when the center of the recurrent tumor existed outside of the planning target volume. Neurotoxicity was suspected by routine physical, neurological and MRI examinations, in which brain atrophy and white matter changes were evaluated according to the method described previously [18]. In general, MRI was performed at 3- or 4-month intervals. Mini–mental state examination was employed for screening. Final diagnosis of neurocognitive dysfunction was made by neurologists using other tests for higher brain function.

### Statistical analysis

Patient, tumor and treatment characteristics and response rates were compared by the Fisher exact test or *t*-test. Rates for overall survival, progression-free survival, central nervous system (CNS) recurrence, and in-field and out-of-field CNS recurrence were calculated by the Kaplan–Meier method from the date of start of radiation therapy. The log-rank test was used to compare the results between pairs of groups. The Cox proportional hazards model was used for multivariate analysis of prognostic factors. A computer program SPSS ver. 23 (IBM, New York, USA) was used for all the statistical analyses.

## RESULTS

**Fig. 1.**
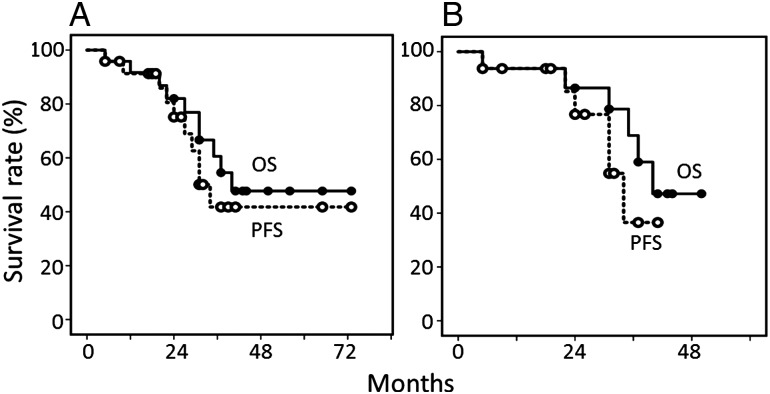
Overall survival (OS) and progression-free survival (PFS) curves for 24 patients undergoing PBRT (**A**) and 16 patients receiving PBRT plus MTX-based chemotherapy (**B**).

**Fig. 2.**
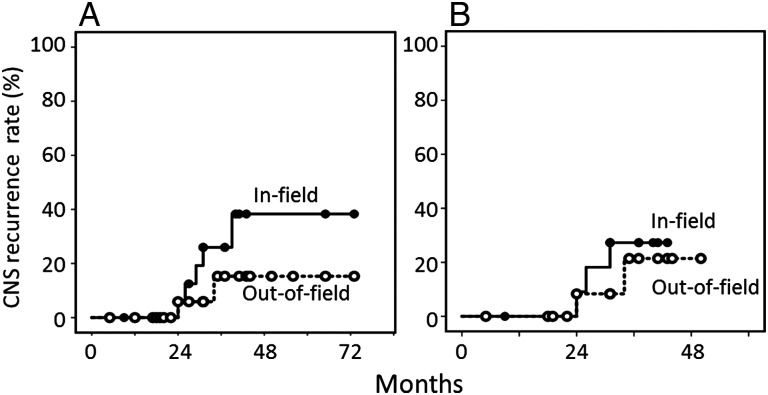
In-field and out-of-field CNS recurrence curves for 24 patients undergoing PBRT (**A**) and 16 patients undergoing PBRT plus MTX-based chemotherapy (**B**).

For the 24 patients undergoing PBRT, the median follow-up period for living patients was 40 months, and the initial response rate was 92%. Figure 1 shows the overall and progression-free survival curves for all 24 patients and 16 patients undergoing PBRT plus MTX-based chemotherapy. The overall survival rates were 60% at 3 years and 47% at 5 years for all 24 patients, and 68% at 3 years for the 16 patients undergoing MTX-based chemotherapy. The progression-free survival rates were 41% at 3–5 years for all 24 patients and 36% at 3 years for the 16 patients undergoing MTX-based chemotherapy. Figure 2 shows the rates for in-field and out-of-field CNS recurrences in patients undergoing PBRT. The in-field recurrence rate was 26% and the out-of-field recurrence rate was 15% at 3 years for all 24 patients. These rates were 27% and 21%, respectively, for the 16 patients undergoing MTX-based chemotherapy.

**Fig. 3.**
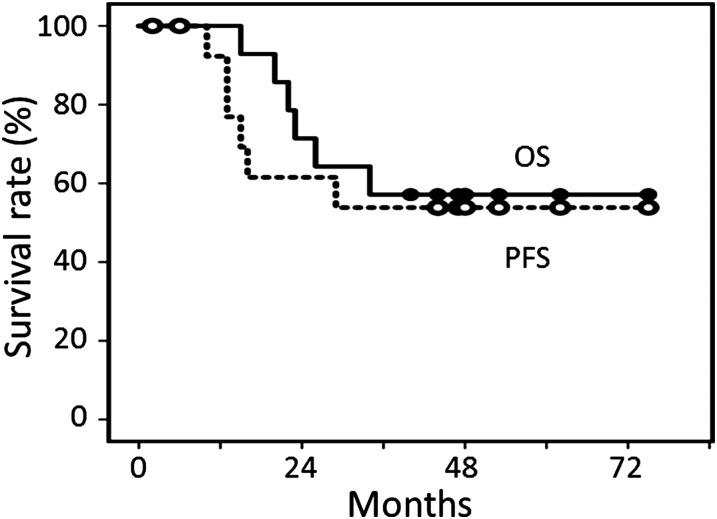
Overall survival (OS) and progression-free survival (PFS) curves for 15 patients undergoing WBRT plus MTX-based chemotherapy.

**Fig. 4.**
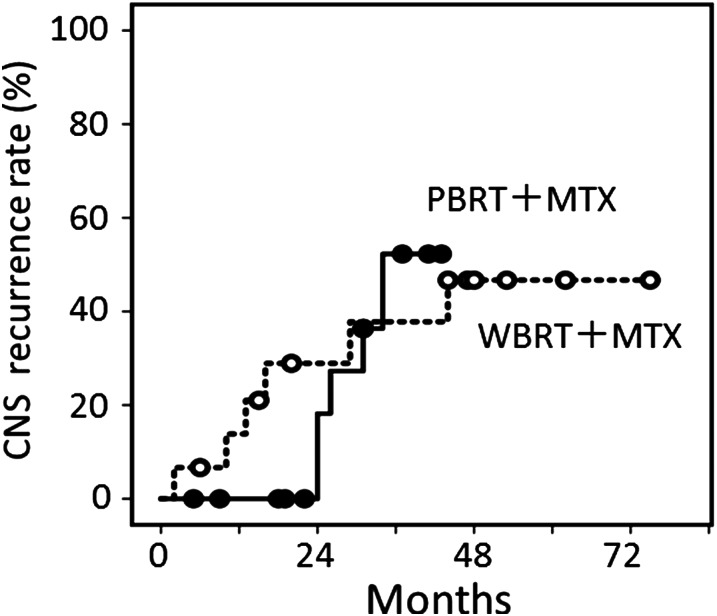
CNS recurrence curves for 16 patients undergoing PBRT plus MTX-based chemotherapy and 15 patients undergoing WBRT plus MTX-based chemotherapy.

For the 15 patients undergoing WBRT plus MTX-based chemotherapy, the median follow-up period for living patients was 40 months, and the initial response rate was 93%. Figure 3 shows the overall and progression-free survival curves for the 15 patients. The overall survival rates were 57% at 3–5 years. The progression-free survival rates were 53% at 3–5 years. Six of the 15 patients developed CNS recurrence, and in two of them, their tumors recurred at sites distant from the primary tumor site. In Fig. 4, the CNS-recurrence rates in patients undergoing MTX-based chemotherapy and PBRT or WBRT are shown.

Evident late neurotoxicity was encountered in 3 of the 16 (19%) patients undergoing PBRT plus MTX-based chemotherapy; 2 were >60 years old and one was 58 years old. None of the 8 patients not undergoing MTX-based chemotherapy developed neurotoxicity. Late neurotoxicity was observed in 4 of the 15 (26%) patients undergoing WBRT plus MTX-based chemotherapy; 3 were >60 years old and 1 was <60 years old. The difference was not significant (*P* = 0.68).

## DISCUSSION

The potential benefits and disadvantages of using PBRT for PCNSL were discussed by Shibamoto *et al.* [17, 19] previously. In their study, they analyzed 43 patients undergoing PBRT collected from many institutions all over Japan; our series should be the largest one investigating PBRT in a single group. The biggest benefit of PBRT may be the sparing of normal brain tissues. Although it is unclear whether PBRT leads to reduced radiation neurotoxicity, it was reported that there was a trend towards a higher and more stable PS, better memory function, and superior employment history in glioma patients treated with PBRT, compared with those receiving WBRT [20]. In the present study, neurocognitive function was not assessed in a prospective fashion, and we could not demonstrate that PBRT was associated with lower neurotoxicity. Thus, this potential benefit needs to be investigated prospectively, but we think that it is, at least, not undesirable to spare normal brain tissues by using PBRT.

The potential disadvantage of PBRT is a concern about out-of-field recurrence. PCSNLs are quite often multiple, and a recent Japanese survey revealed that 52% of PCNSLs were multiple on diagnostic imaging [8]. This indicates that PCNSL may occur at multiple CNS sites, even when the lesion appears to be singular; this may contraindicate the use of PBRT. On the other hand, multiple contrast-enhancing lesions are often thought to be contiguous, with hypointense and hypodense areas on T1-weighted MR and CT images, respectively, between the separate contrast enhancements. Therefore, we used PBRT even in patients with multiple contrast-enhancing lesions when they were considered contiguous. As a result, the out-of-field recurrence rate was 15% at 3 years in patients undergoing PBRT. We are not sure whether this out-of-field recurrence rate is acceptable or not, but PCNSL can recur at CNS sites distant from the primary tumor, even after WBRT [17]. Also in the present study, 2 of the 15 patients undergoing WBRT + MTX-based chemotherapy developed CNS recurrence at sites distant from the primary tumor. Recurrence patterns after PBRT should be further investigated to determine whether PBRT is an acceptable treatment or not.

Since the introduction of high-dose-MTX–based chemotherapy, the use of radiation therapy has decreased, and the omission of radiation in first-line treatment has been attempted, even in non-elderly patients [21, 22]. However, the omission of radiation therapy has led to higher recurrence rates and lower cure rates. Indeed, many long-term survivors who underwent conventional doses of radiation therapy [23, 24] have been documented in the literature. How to clarify the optimal use of radiation therapy in combination with chemotherapy is the next topic to be investigated. Use of radiation doses lower than have been conventionally used (50 Gy) should be investigated, as is recommended in a recently published European guideline [25], but tumor mass regions may require relatively high doses. PBRT should be an attractive mode of radiation therapy, but out-of-field recurrence is always a concern. After fully taking these issues into consideration, we recommend the use of rotational intensity-modulated radiation therapy (i.e. helical tomotherapy or volumetric-modulated arc therapy) for PBRT. With these techniques, a simultaneous integrated boost to the tumor can readily be delivered. In addition, some doses are uniformly delivered around the PTV, and dose fall-off around the PTV is not so steep. These low doses may have some impact on decreasing out-of-field recurrence. We are now using helical tomotherapy for all patients with PCNSL undergoing PBRT.

In conclusion, PBRT appears to yield acceptable overall and progression-free survival in PCNSL patients. Incorporation of PBRT into prospective trials for PCNSL should be considered.

## FUNDING

Funding to pay the Open Access publication charges for this article was provided by Department of Radiology, Nagoya City University Graduate School of Medical Sciences.
